# Mesenchymal stromal cell treatment prevents H9N2 avian influenza virus-induced acute lung injury in mice

**DOI:** 10.1186/s13287-016-0395-z

**Published:** 2016-10-28

**Authors:** Yan Li, Jun Xu, Weiqing Shi, Cheng Chen, Yan Shao, Limei Zhu, Wei Lu, XiaoDong Han

**Affiliations:** 1 Department of Chronic Communicable Disease, Jiangsu Provincial Center for Disease Prevention and Control, Nanjing, 210009 People’s Republic of China; 2 Medical School, Nanjing University, Nanjing, Jiangsu 210093 People’s Republic of China; 3 Institute of Toxicology & Functional Assessment, Jiangsu Provincial Center for Disease Prevention and Control, Nanjing, 210009 People’s Republic of China

**Keywords:** Mesenchymal stromal cell, H9N2 avian influenza viruses, Lung injury, Cell therapy

## Abstract

**Background:**

The avian influenza virus (AIV) can cross species barriers and expand its host range from birds to mammals, even humans. Avian influenza is characterized by pronounced activation of the proinflammatory cytokine cascade, which perpetuates the inflammatory response, leading to persistent systemic inflammatory response syndrome and pulmonary infection in animals and humans. There are currently no specific treatment strategies for avian influenza.

**Methods:**

We hypothesized that mesenchymal stromal cells (MSCs) would have beneficial effects in the treatment of H9N2 AIV-induced acute lung injury in mice. Six- to 8-week-old C57BL/6 mice were infected intranasally with 1 × 10^4^ MID_50_ of A/HONG KONG/2108/2003 [H9N2 (HK)] H9N2 virus to induce acute lung injury. After 30 min, syngeneic MSCs were delivered through the caudal vein. Three days after infection, we measured the survival rate, lung weight, arterial blood gas, and cytokines in both bronchoalveolar lavage fluid (BALF) and serum, and assessed pathological changes to the lungs.

**Results:**

MSC administration significantly palliated H9N2 AIV-induced pulmonary inflammation by reducing chemokines and proinflammatory cytokines levels, as well as reducing inflammatory cell recruit into the lungs. Thus, H9N2 AIV-induced lung injury was markedly alleviated in mice treated with MSCs. Lung histopathology and arterial blood gas analysis were improved in mice with H9N2 AIV-induced lung injury following MSC treatment.

**Conclusions:**

MSC treatment significantly reduces H9N2 AIV-induced acute lung injury in mice and is associated with reduced pulmonary inflammation. These results indicate a potential role for MSC therapy in the treatment of clinical avian influenza.

## Background

Infections with avian influenza virus (AIV) strains have become highly prevalent in poultry worldwide [1–4]. Avian influenza (AI) is a leading cause of morbidity, mortality, and economic loss in many countries. Mammals can also be infected with several AIV subtypes, including H5N1, H9N2, H7N7, and H7N3 [5, 6]. In 1997, 18 people were infected with avian H5N1 influenza virus and six died, refocusing global attention on the potential role of AIVs as precursors of human pandemic influenza virus strains [7, 8]. The H9N2 strain has been isolated from pigs and humans with influenza-like illnesses in Hong Kong and mainland China since 1998 [9, 10]. These findings indicate that the AIV can also cross species barriers and expand its host range from birds to mammals, thus highlighting the pandemic potential of the H9N2 virus.

Although the H9N2 avian virus subtype is generally not highly pathogenic for avian species, it has been associated with severe morbidity and mortality in poultry following coinfection with other pathogens [11, 12]. Recent findings indicate that the H5N1 viruses responsible for severe human disease contain genetic rearrangements that include several genes from obtained avian H9N2 viruses [13].

However, despite decades of research, few therapeutic strategies for clinical AI have emerged, and current specific treatment options are limited. Treatment of AI currently still relies on antiviral agents. A recent Cochrane review reported that antiviral therapies had little benefit for severe influenza patients [14, 15]. Furthermore, AI continues to require prolonged mechanical ventilation in the intensive care unit, and AI-associated mortality remains high at 30–50 % despite optimal supportive care [3, 16].

The reasons for the high mortality associated with AI are unknown; the presence of a new viral subtype to which the human host has no prior immunity cannot totally explain this phenomenon. AIV is well known to mainly cause pneumonia, severe acute lung injury (ALI), and acute respiratory distress syndrome (ARDS) in humans [17]. A clinically important pronounced activation of the proinflammatory cytokine cascade perpetuates the inflammatory response and may contribute to further tissue damage and persistence of the systemic inflammatory response syndrome, leading to pulmonary infection in both animals and humans [18, 19]. We therefore hypothesized that improving immune regulation would benefit the treatment of AI.

Cell therapies using bone marrow-derived mesenchymal stromal cells (MSCs) have emerged as potential novel therapeutic approaches for several diseases [20, 21]. Several studies have shown that both ectogenic and endogenic MSCs can migrate into the lung, adopt the phenotype of lung cells, and play positive roles in repairing lung injury, including that caused by ARDS, emphysema, and idiopathic pulmonary fibrosis [22–24]. Importantly, recent studies have demonstrated that bone marrow-derived MSCs can exhibit immunosuppressive properties. In addition, MSCs have been suggested to be “immune evasive”, and thus protected from rejection, which potentially permits their use in allotransplantation [25, 26]. MSCs may also engraft in the injured lung and can even differentiate into lung epithelial cells in vivo [27]. Therefore, MSCs may be beneficial in the treatment of AI.

The aim of this study was to evaluate the effect of MSC treatment on lung inflammation and injury induced by H9N2 AIV infection in a murine model.

## Methods

### Isolation, culture, and characterization of MSCs

MSCs were isolated from the bone marrow of C57BL/6 mice weighing 20–30 g, obtained from the Shanghai Laboratory Animal Research Center (Shanghai, China) and maintained under specific pathogen-free conditions. Bone marrow was flushed from the femurs under aseptic conditions using DMEM. The collected cells were washed three times with PBS, resuspended in DMEM containing 10 % fetal bovine serum and 1 % l-glutamine, and seeded at 1 × 10^6^ cells/ml into culture flasks. Cells were maintained in a humidified atmosphere of 95 % air and 5 % CO_2_ at 37 °C. Nonadherent cells were discarded after 48 h and the culture medium changed every 3–4 days thereafter. Cells were harvested when they reached approximately 90 % confluency and were diluted 1:2 or 1:3 at each passage. MSCs used in all in vivo experiments were between passages 3 and 10.

### Fluorescence-activated cell sorting analysis

Passage 3–10 MSCs were analyzed for the following markers: CD34, CD45, CD73, CD79, CD90, and CD105. A total of 1 × 10^5^ cells were incubated with each fluorescence-conjugated primary antibody at 37 °C for 2 h in the dark. After three PBS washes, cells were processed by flow cytometry (BD FACSCalibur) and data were analyzed using Cell Quest software. Antibodies used were: anti-mouse CD34, CD45, and CD79 (Santa Cruz Biotechnology Inc.); anti-mouse CD73, CD90, and CD105 (BioLegend, San Diego, CA), and all of the antibodies were labeled with FITC.

### Differentiation of MSCs

Passage 5 MSCs were differentiated into adipocytes, chondrocytes, and osteocytes using the Mouse Mesenchymal Stem Cell Functional Identification Kit (R&D, USA) to prove their ability to differentiate into multiple mesenchymal lineages. Methods were according to the procedures of protocol; briefly, MSCs were plated at 1 × 10^4^/well in 24-well plates and incubated in α-MEM containing 10 % fetal bovine serum and 1 % l-glutamine until they reached approximately 50–70 % confluency for differentiation. Cells were cultured in adipogenic, osteogenic, or chondrogenic media for 10–21 days before being prepared for lineage-specific immunocytochemistry stains. Adipocytic differentiation was confirmed by staining with FABP4, differentiation to osteocytes was confirmed by staining with osteopontin, and differentiation to chondrocytes was confirmed by staining with collagen II as previously described. Briefly, cells were washed with PBS and fixed in 4 % paraformaldehyde in PBS for 20 min. The cells were permeabilized and blocked with 0.5 ml PBS containing 0.3 % Triton X-100 and 1 % BSA at room temperature for 30 min. After blocking, the cells were incubated with Goat Anti-mouse FABP4, osteopontin, or Sheep Anti-mouse Collagen II antibody working solution for 1 h at 37 °C; they were then washed and incubated with Alexa 555 labelled Rabbit Anti-Goat secondary antibody in the dark for 60 min at room temperature. Images of the stained cells were obtained by using a phase fluorescence microscope (Axio Observer, Zeiss, Germany).

### Murine H9N2 infected mice of H9N2 AIV-induced acute lung injury

A/Hong Kong/2108/2003 [H9N2 (HK)] AIV was kindly provided by Prof. Zheng Xing (Medicine School, Nanjing University). To assess pathological changes induced by H9N2 AIV, 6-week-old female SPF C57BL/6 mice were housed in microisolator cages in the animal facility of the Jiangsu Provincial Center for Disease Prevention and Control under conditions of negative pressure and ventilated with HEPA-filtered air.

A total of 95 C57BL/6 mice were divided into seven groups: A: controls (*n* = 10); B: MSCs (*n* = 10); C: H9N2 infected mice + physiological saline (*n* = 15); D: H9N2 infected mice + McCoy (*n* = 15); E: H9N2 infected mice + MSCs (*n* = 15); F: H9N2 infected mice + physiological saline (*n* = 15); G: H9N2 infected mice + MSCs (*n* = 15). Groups B–D had MSCs, McCoy, or saline administered 30 min following viral infection induction, while groups E and F were administered 1 day after. To induce lung injury in H9N2-infected mice, diethyl ether was used to anesthetize the mice and 1 × 10^4^ MID_50_ (Median Infective Dose) of A/HONG KONG/2108/2003 [H9N2 (HK)] H9N2 AIV dissolved in 10 μl sterile physiological saline was administered intranasally. Control mice were treated with noninfectious allantoic fluid of the equivalent dilution. Thirty minutes after H9N2 AIV challenge, physiological saline, McCoy, or syngeneic MSCs (1 × 10^5^ cells; 100 μl total volume) were slowly infused into each mouse via the caudal vein. Naive mice (without H9N2 AIV instillation) were injected with saline or MSCs to serve as controls for any inflammatory response that might result from the injected MSCs. Mice were humanely killed by ether anesthesia followed by percutaneous left ventricular bleeding 3 days after MSC treatment and their tissues were harvested for analysis. McCoy was used as a negative control.

### Virus titration in lung of mice

Virus titration in lung homogenate of three mice per group was detected to quantify the infection level. Tissues were collected and homogenized in cold phosphate-buffered saline on day 3 after treatment. Clarified homogenates were titrated for viral infectivity in embryonated chicken eggs from initial dilutions of 1:2. Viral titers were expressed as mean log10 EID_50_/ml ± SD (1 MID_50_ is around 1 × 10^3^ EID_50_).

### Survival rate and lung weight

Three days after treatment, the survival rate was observed for each experimental group. In addition, the upper right lung lobes were weighed before and after oven desiccation at 70 °C to determine the lung wet:dry weight ratio. Lung wet:dry weight ratio = weight of the wet lung/weight of the dry lung; relative lung weight (%) = weight of the whole wet lung/body weight × 100 %. These were used as indicators of lung edema.

### Histopathology

Lung tissue samples of three mice per group (one cross section of the right lower lobe, one of the right mid lobe, and one of the right upper lobe) were fixed in 4 % paraformaldehyde, embedded in paraffin, and cut into 4-μm thick sections. Sections were stained with hematoxylin and eosin, and observed using an optical microscope. All lungs were uniformally inflated and fixed in the same way. The average interalveolar septum thickness was quantified in a blinded fashion by measuring the thickness of all septae along a crosshair placed on each image (at least 100 septae were measured per animal). Lung injury score was observed for quantification of the lung damage; briefly, images were evaluated by an investigator who was blinded to the identity of the slides (WCL) according to a previously defined scoring system [28]. The grading system was as follows: 0, minimal damage; 1, mild damage; 2, moderate damage; 3, severe damage; and 4, maximal damage.

### Arterial blood gas analysis

Five mice from each group were anesthetized with diethyl ether on day 3 postinoculation. Arterial blood samples (100 μl) of lightly anesthetized mice spontaneously breathing room air were withdrawn into a heparinized syringe by percutaneous left ventricular sampling. Blood gas analysis was immediately performed with an i-STAT 300 blood gas/electrolytes analyzer (Abbott, USA).

### Cytokine measurement

Concentrations of the chemokines granulocyte-macrophage colony-stimulating factor (GM-CSF), monocyte chemoattractant protein (MCP-1), keratinocyte chemoattractant (KC), macrophage inflammatory protein-1α (MIP-1α), and monokine induced by IFN-γ (MIG) and the inflammatory cytokines interleukin (IL)-1α, IL-6, IL-10, tumor necrosis factor alpha (TNF-α), and interferon (IFN-γ) in bronchoalveolar lavage fluid (BALF) and serum (five mice from each group) were measured 3 days after H9N2 infection using the Mouse Cytokine Magnetic 20-Plex Panel (Invitrogen) by Luminex 100 (Bio-Rad, USA).

BALF of mice was collected immediately following sacrifice. Briefly, the lungs were lavaged three times with a total volume of 1.0 ml physiological saline (4 °C) in the chest cavity opened by midline incision. The rate of recovery of BALF was not less than 90 % for all of the animals tested.

### Protein expression

Expression of CD14, TLR4, ERK, and JNK protein in lung tissue was measured 3 days after H9N2 infection using Western blotting. Lung tissue proteins were obtained from the right lower lungs of mice in each group. Briefly, tissues were lysed in ice-cold extraction buffer containing protease inhibitor cocktail (Roche) and then centrifuged at 12,000 g for 30 min; the protein concentration in the supernatant was determined using BCA assays. Proteins were separated using 10 % SDS-polyacrylamide gel electrophoresis and electrophoretically transferred to polyvinylidene fluoride (PVDF) membranes using standard procedures. The membranes were blocked at 37 °C for 1 h in phosphate-buffered saline (PBS) containing 0.05 % (vol/vol) Tween 20 and 5 % (wt/vol) non-fat milk for 1 h at RT. Membranes were then incubated in primary antibody at a 1:500 dilution in PBSTM (PBS containing 0.05 % (vol/vol) Tween 20 and 0.1 % (wt/vol) non-fat milk) for 3 h at RT and then washed three times for 5 min each in PBSTM. Membranes were then incubated with the appropriate horseradish peroxidase (HRP)-conjugated anti-species secondary antibodies (Boster, Wuhan, China) for 1 h at RT and then washed in PBSTM as described above. Immunoreactive protein bands were detected using an Odyssey Scanning System (LI-COR, Inc., Lincoln, NE, USA). Ratios for the protein of interest (POI) were expressed relative to GAPDH in the same sample as a loading control. The primary antibodies rabbit anti-CD14, TLR4, ERK, JNK, and GAPDH were obtained from Santa Cruz (USA).

### Ethics Statement

This study was carried out in strict accordance with the recommendations in the Guide for the Care and Use of Laboratory Animals of the National Institutes of Health. The protocol was approved by the Committee on the Ethics of Animal Experiments of the University of Minnesota (approval no. A9089). All surgery was performed under sodium pentobarbital anesthesia, and all efforts were made to minimize suffering.

### Statistical analysis

Data are expressed as the mean ± standard deviation (S.D.). All calculations and statistical analyses were performed using SPSS for windows version 13.0 (SPSS Inc., Chicago, IL, USA). One-way ANOVA followed by Dunnett’s *t* test was used to analyze differences between groups. *P* < 0.05 was regarded as statistically significant.

## Results

### Highly pure MSCs can be isolated from murine bone marrow

**Fig. 1:**
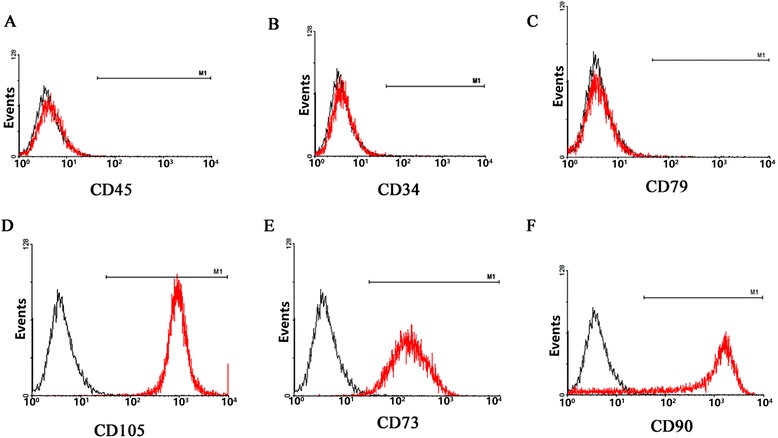
Flow cytometric analysis of MSCs. Isolated MSCs do not express CD45, CD34, or CD79 (**a**–**c**), but do express C105, CD73, and CD90 (**d**–**f**). *Grey* lines represent a non-stained control, *red* lines represent specific antibody staining

**Fig. 2:**
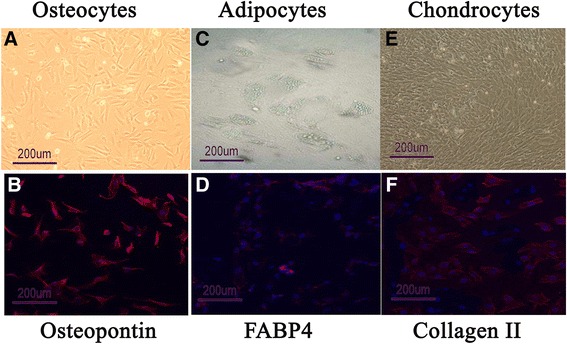
Differentiation of passage 5 MSCs into osteocytes, adipocytes, and chondrocytes. Fluorescence microscopy images of osteogenic differentiation (osteopontin staining of undifferentiated MSCs (**a**) and differentiated osteocytes (**b**)), adipogenic differentiation (FABP4 staining of undifferentiated MSCs (**c**), and differentiated adipocytes (**d**)), and chondrogenic differentiation (collagen II of undifferentiated MSCs (**e**) and differentiated chondrocytes (**f**))

After approximately 2 weeks of culture, isolated MSCs had expanded significantly and exhibited evident plastic adherence. After three to four passages, MSCs exhibited a homogeneous fibroblast-like, spindle-shaped morphology. FACS analysis demonstrated that passage 3–10 MSCs did not express CD34, CD45, or CD79 (Fig. 1a–c), but did express CD73, CD90, and CD105 (Fig. 1d–f). These data indicate that the cultured cells were of mesenchymal origin and of a high purity. Passage 5 MSCs readily differentiated into adipocytes, osteocytes, or chondrocytes when incubated in differentiation medium, confirming their pluripotent potential (Fig. 2).

### Virus titration in the lungs of mice

**Fig. 3:**
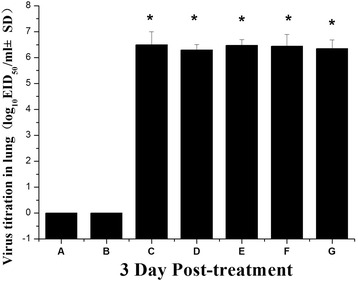
Virus titration in the lungs of mice. Mean viral titers based on three mice per group are expressed as log_10_ EID_50_ per milliliter ± SD

H9N2 viral infection resulted in high viral titers in the lungs; the viral titers of the H9N2 infected mice + physiological saline group, H9N2 infected mice + McCoy group, and H9N2 infected mice + MSCs group exceeded 6.0 log_10_ EID_50_/ml (Fig. 3) on day 3 post-infection.

### Survival rate and lung weight

**Fig. 4:**
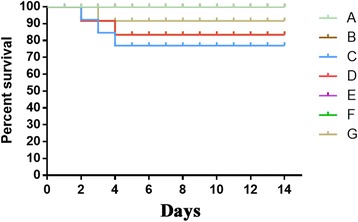
The survival rate of mice in different experimental groups 3 days post-treatment. Kaplan-Meier curves are shown for mice in different groups. MSCs could improve survival in experimental AI mice. See text for definition of groups A–G

Three days after infusion of MSCs, we observed that the survival rate of mice in the H9N2 infected mice + MSCs group was higher than that of the H9N2-infected mice groups; the survival rate of the H9N2 infected mice + MSCs group was 100 %, whereas that of the H9N2-infected mice groups were approximately 80 % (3 deaths out of 15 mice in the H9N2 infected mice + physiological saline group and 2 deaths out of 15 mice in the H9N2 infected mice + McCoy groups) (Fig. 4).

**Fig. 5:**
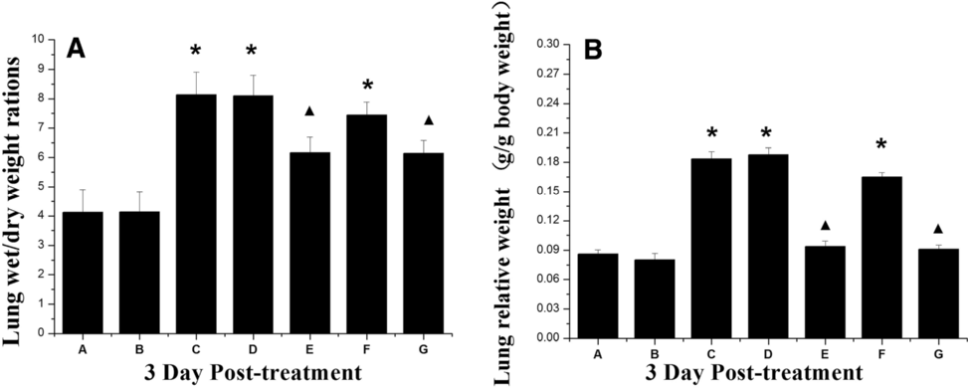
Effect of MSCs on lung weights of mice in comparison to control mice. **a** Lung wet:dry weight ratios and **b** relative lung weight. *Response that is significantly different from the control (*p* < 0.05).^▲^Response that is significantly different from the H9N2 infected mice (*p* < 0.05). See text for definition of groups A–G

Mean relative lung weights and lung wet:dry weight ratios were significantly higher (*p* < 0.05) in H9N2-infected lungs versus control mice and significantly lower (*p* < 0.05) in mice treated with H9N2 + MSCs versus those of the H9N2-infected groups (Fig. 5).

### Histopathology

**Fig. 6:**
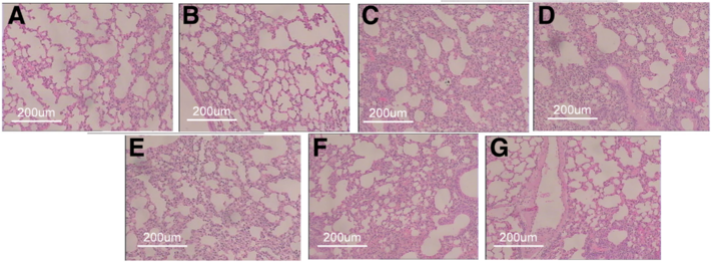
Histological evaluation of the therapeutic potential of MSCs on AIV-induced lung injury in mice. Lung pathology of mice in different experimental groups 3 days post-treatment (hematoxylin and eosin, 100× magnification). **a** Control group, **b** MSC group (lungs of control and MSC groups showed a normal aspect), **c** H9N2 infected mice + physiological saline group, **d** H9N2 infected mice + McCoy group (diffuse pneumonia and severe alveolar injury were observed), **e** H9N2 infected mice + MSCs group (MSC transplantation reduced lung injury). **f** H9N2 infected mice+ Physiological saline administer 1 day following viral infection induction (Severe diffuse pneumonia and extensive alveolar damage). **g** H9N2 infected mice+MSCs administer 1 day following viral infection induction (MSC transplantation reduced lung inflammation and injury)

**Fig. 7:**
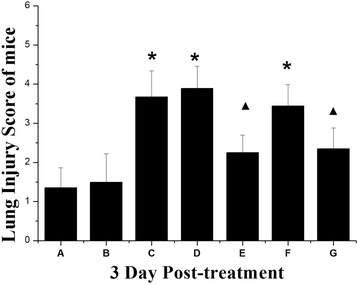
Lung injury score of mice in different experimental groups 3 days post-treatment. Data are represented as mean ± standard error of the mean; *n* =6 per group. *Response that is significantly different from the control (*p* < 0.05). ^▲^Response that is significantly different from the H9N2 infected mice (*p* < 0.05). See text for definition of groups A–G

Lung sections from mice in the H9N2 infected mice + physiological saline group and H9N2 infected mice + McCoy group displayed similar histopathological patterns consistent with severe diffuse pneumonia and characterized by inflammatory cellular infiltration, numerous polymorphonuclear leukocytes and macrophages in interstitial spaces, interstitial and alveolar edema, and hemorrhage. Diffuse pneumonia with severe alveolar damage was observed throughout the entire lung (Fig. 6c and d). Administration of MSCs (H9N2 infected mice + MSCs) reduced airspace inflammation and improved lung histopathology. Lung structure was less abnormal than in the H9N2 infected mice + physiological saline group, suggesting that therapy with MSCs is protective against H9N2-induced lung injury (Fig. 6e). The severity of lung injury was also assessed using a semiquantitative histopathological scoring system that evaluated lung injury in four categories: alveolar septae, alveolar hemorrhage, intra-alveolar fibrin, and intra-alveolar infiltrates. Although treatment with MSCs tended to reduce lung injury scores (Fig. 7), the observed differences did not reach statistical significance.

### Arterial blood gas analysis

Arterial blood gas parameters in the different experimental groups are shown in Table 1. In the H9N2-infected mice, the partial pressure of arterial oxygen (PaO_2_) was significantly lower, while the partial pressure of arterial carbon dioxide (PaCO_2_) was significantly higher, and the values for saturation of arterial oxygen (SaO_2_) and pH were slightly lower. Compared with the H9N2 infected mice + physiological saline and H9N2 infected mice + McCoy groups, mice in the H9N2 infected + MSCs group had significantly higher PaO_2_, significantly lower PaCO_2_, and slightly higher (but still statistically significantly different) SaO_2_ and pH values. These results indicate that most of the H9N2 AIV infected mice developed severe hypoxemia, and that MSCs effectively protected against functional pulmonary injury.

**Table 1:** Effect of H9N2 avian influenza virus (AIV) and mesenchymal stromal cells (MSCs) on arterial blood gas analysis of mice

Groups	pH	PO_2_ (mmHg)	PCO_2_ (mmHg)	SO_2_
A: Control (allantoic fluid; *n* = 10)	7.23 ± 0.032	90.8 ± 6.43	38.5 ± 1.66	92.7 ± 0.73
B: MSCs (1 × 10^5^ MSCs; *n* = 10)	7.29 ± 0.031	88.2 ± 5.51	37.9 ± 1.11	93.8 ± 1.02
C: H9N2-infected mice (1 × 10^4^ MID_50_ H9N2AIV; *n* = 15)	6.62 ± 0.286	46 ± 4.17*	65.34 ± 2.55*	64.82 ± 1.63*
D: H9N2-infected mice + MSCs (1 × 10^4^ MID_50_ H9N2AIV + 1 × 10^5^ McCoy; *n* = 15)	6.71 ± 0.257	48.2 ± 5.11*	64.32 ± 1.98*	68.15 ± 1.75*
E: H9N2-infected mice + MSCs (1 × 10^4^ MID_50_ H9N2AIV + 1 × 10^5^ MSCs; *n* = 15, 30 min following infection)	6.84 ± 0.613	68 ± 5.76*^▲^	50.05 ± 1.09*^▲^	78.47 ± 1.54*^▲^
F: H9N2-infected mice (1 × 10^4^ MID_50_ H9N2AIV; *n* = 15)	6.97 ± 0.375	46 ± 4.35*	66 ± 3.34*	65 ± 2.44*
G: H9N2-infected mice + MSCs (1 × 10^4^ MID50 H9N2AIV + 1 × 10^5^ MSCs; *n* = 15, 1 day following infection)	6.77 ± 0.712	60 ± 3.35*^▲^	52.39 ± 4.13*^▲^	75.89 ± 2.44*^▲^

*Response that is significantly different from the control (*p* < 0.05)

^▲^Response that is significantly different from the H9N2-infected mice (*p* < 0.05)

*PaCO*_*2*_ partial pressure of arterial carbon dioxide, *PaO*_*2*_ partial pressure of arterial oxygen, *SaO*_*2*_ saturation of arterial oxygen

### Cytokine measurements

#### Chemokines

**Fig. 8:**
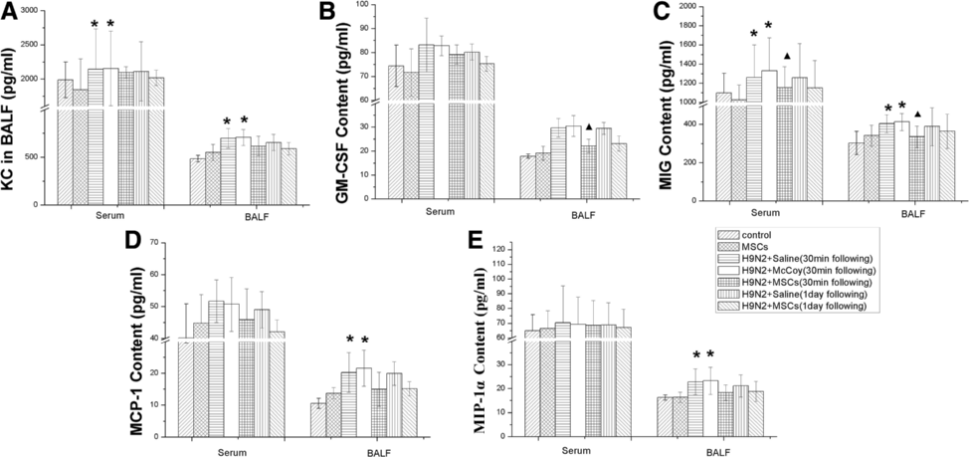
Levels of chemokines in bronchoalveolar lavage fluid (*BALF*) and serum. Concentrations of the chemokines granulocyte-macrophage colony-stimulating factor (*GM-CSF*), monocyte chemoattractant protein (*MCP-1*), keratinocyte chemoattractant (*KC*), macrophage inflammatory protein-1α (*MIP-1α*), and monokine induced by IFN-γ (*MIG*) in each group were measured using Luminex in bronchoalveolar lavage fluid and serum. Groups as legend. Group comparisons were made using one-way ANOVA with Dunnett’s post hoc test. *p < 0.05; **p < 0.01; *n* = 6 mice per group. *MSC* mesenchymal stromal cell

Three days after H9N2 infection, the concentrations of GM-CSF, MCP-1, KC, MIP-1α, and MIG were measured in both BALF and serum. The concentrations of all chemokines were increased in BALF and serum (Fig. 8) in the H9N2 infected mice + physiological saline group. In contrast, the concentrations of chemokines were much lower in the H9N2 infected mice + MSCs group.

#### Inflammatory cytokines

**Fig. 9:**
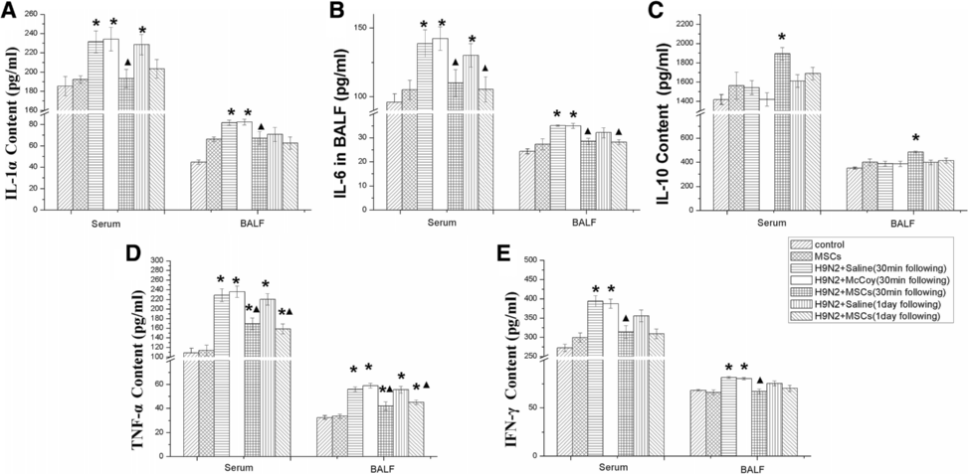
Levels of inflammatory cytokines in bronchoalveolar lavage fluid (*BALF*) and serum. Concentrations of the inflammatory cytokines interleukin (*IL*)-1α, IL-6, tumor necrosis factor alpha (*TNF-α*), and interferon gamma (IFN-γ), and of IL-10 were measured using Luminex in bronchoalveolar lavage fluid and serum. Groups as legend. Group comparisons were made using one-way ANOVA with Dunnett’s post hoc test. *p < 0.05; **p < 0.01; n = 6 mice per group. *MSC* mesenchymal stromal cell

The concentrations of IL-1α, IL-6, IL-10, TNF-α, and IFN-γ were measured in BALF and serum 3 days after H9N2 infection. In the H9N2 infected mice + physiological saline group, the concentrations of these inflammatory cytokines were significantly increased in both BALF and serum (Fig. 9). In contrast, the concentrations were significantly lower in the H9N2 infected mice + MSCs group.

### Protein expression

**Fig. 10:**
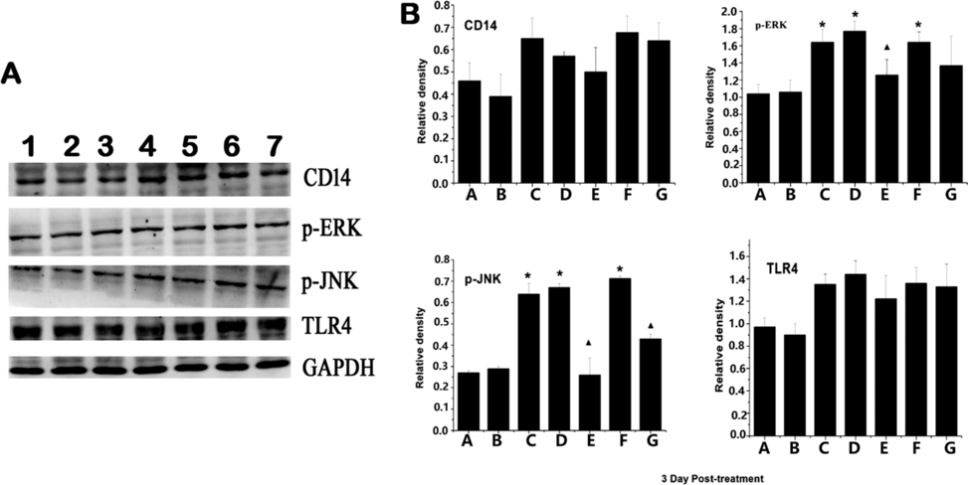
Western blotting analyzed the expression of TLR4-related protein in the lung tissue after MSC transplantation for 3 days. **a** Expression of CD14, TLR4, ERK, and JNK were evaluated by immunoblotting using specific antibodies. GAPDH is used as the control. 1: controls, 2: MSCs, 3: H9N2, 4: H9N2 + McCoy, 5: H9N2 + MSCs, 6: H9N2 + Physiological saline (1 day following) 7: H9N2 +MSCs (1 day following). **b** Responses were quantified by densitometry and normalized to the expression of GAPDH. Densitometry data are shown as mean ± SD. *Response that is significantly different from the control (*p* < 0.05). ^▲^Response that is significantly different from the H9N2 infected mice (*p* < 0.05)

Three days after H9N2 infection, the expression of CD14, TLR4, ERK and JNK protein in the lung tissue was measured. In the H9N2-infected mice, the expression of CD14, TLR4, ERK, and JNK protein in lung tissue was significantly increased compared with the control group. Mice in the H9N2 infected + MSCs group had significantly decreased ERK and JNK expression compared with the H9N2 infected mice + physiological saline and H9N2 infected mice + McCoy groups. The expression of CD14 and TLR4 in mice in the H9N2 infected + MSCs group tended to be lower, although not significantly so (Fig. 10).

## Discussion

The present study demonstrated that MSCs exert a beneficial therapeutic effect on H9N2 AIV-induced lung injury in a murine model; treatment with MSCs results in improvements in both pulmonary inflammation and lung tissue organization. These findings have potentially important implications for the treatment of AI, which is an important clinical problem characterized by high patient morbidity and mortality.

Avian H9N2 viruses have been widespread in domestic poultry in Asian countries since the mid-1990s, with a mortality ranging from 5–30 % [29, 30]. Many studies have shown that H9N2 viruses can cause upper respiratory tract illnesses in humans. This indicates that the virus has evolved to cross the species barrier and is capable of infecting humans, bypassing intermediate hosts.

H9N2 viruses can activate the innate immune response. A hallmark of pulmonary infiltration associated with AI is the presence of infiltrating leukocytes [31, 32]. Leukocyte migration is directed largely by chemokines, and the inter-relationship of early-response cytokines, adhesion molecules, and chemokines orchestrates the recruitment of neutrophils into the lungs [33]. In most studies, cytokines have been described as forming an inflammatory “cascade” or “network” in patients [30, 34]. Lung injury may be a direct consequence of this inflammatory response.

Adult stem cells or progenitor cells are being evaluated for the treatment of a number of diseases that currently have limited or no treatment options [35, 36]. Marrow-derived stem cells are hypothesized to be the source of lung regeneration and repair. An alternative source are exogenous stem/progenitor cells, delivered into the lung either intravenously via the trachea or by direct injection. Recently, many studies have confirmed that MSCs can engraft in the injured lung [37–39] and can even differentiate into lung epithelial cells in vivo. MSCs may also exhibit immunosuppressive properties, suggesting “immune-privilege”, and may even have certain immune regulation functions [40–42]. Therefore, MSCs may, in their own right, have beneficial effects in ALI.

Our data showed that H9N2 viral infection dramatically increases the expression of chemokines, including GM-CSF, MCP-1, KC, MIP-1α, and MIG, in both BALF and serum. However, MSC treatment decreases the expression of these chemokines. We also showed that MSC-treated mice have significantly reduced levels of some inflammatory cytokines (IL-1α, IL-6, TNF-α, and IFN-γ) and a corresponding increase in anti-inflammatory cytokines (IL-10). It is believed that IL-1α, IL-6, TNF-α, and IFN-γ play important roles in the development of ALI [43]. These results are consistent with several previous studies. For example, in Mei’s study, treatment with MSCs alone significantly reduced LPS-induced acute pulmonary inflammation in mice [39]. Importantly, our in vivo experimental results suggest that the administration of MSCs can greatly improve the hypoxemia and histopathological changes of lung injury induced by H9N2 AIV infection. The data indicate that administration of MSCs results in a marked increase in survival rates, primarily due to a decrease in ALI. These results are not consistent with the findings of Lam et al. [44], who reported that MSC therapy fails to improve outcomes in experimental HIN1 influenza. We speculate that this might be a consequence of different pathogenic characteristics of different influenza viruses. It is known that avian influenza virus infection can lead to a “cytokine storm”. In consequence, the host cellular response may be different from that with H1N1 influenza virus infection.

We have, therefore, demonstrated that MSC-based cell therapy can attenuate the inflammatory reaction and injury in the lungs caused by H9N2 AIV exposure, as well as by reducing lung histopathological changes. These beneficial effects may be mediated by the downregulation of chemokines such as GM-CSF, MCP-1, KC, MIP-1α, and MIG, leading to the downregulation of inflammatory cytokines such as TNF-α, IL-1α, IL-6, and IFN-γ. Our findings provide further support for a prominent role of TLR4-dependent immune regulation in acute lung injury therapy. The quantity of stem cells used in our study was 1 × 10^5^. This is much less than used by Wang et al. [45]. We did not find an obvious stem cell niche in the lung tissue of mice. Therefore, we deduce that the immune regulation function of stem cells is via a paracrine mechanism.

On the other hand, it is possible that MSCs may be involved in the early stages of carcinogenesis through spontaneous transformation. In addition, it has been suggested that MSCs can modulate tumor growth and metastasis, although this remains controversial and poorly understood. Interestingly, different studies have reported contradicting findings, with some finding that MSCs promote tumor growth and others that they inhibit tumor growth. Therefore, the role of MSCs in avian influenza infection requires ongoing surveillance.

## Conclusions

Our results suggest that MSC-based therapy can reduce inflammatory lung injury and pulmonary vascular permeability. This may provide a novel strategy for the treatment of AIV-induced lung injury through improving tissue repair and protecting against inflammation. Further investigations into the application of MSC-based cell therapy may give new hope for patients with AIV-induced ALI.

## Abbreviations

AI: Avian influenza
AIV: Avian influenza virus
ALI: Acute lung injury
ARDS: Acute respiratory distress syndrome
MSC: Mesenchymal stromal cell
